# Short-Chain 3-Hydroxyacyl-Coenzyme A Dehydrogenase Associates with a Protein Super-Complex Integrating Multiple Metabolic Pathways

**DOI:** 10.1371/journal.pone.0035048

**Published:** 2012-04-09

**Authors:** Srinivas B. Narayan, Stephen R. Master, Anthony N. Sireci, Charlene Bierl, Paige E. Stanley, Changhong Li, Charles A. Stanley, Michael J. Bennett

**Affiliations:** 1 Department of Pathology and Laboratory Medicine, Children's Hospital of Philadelphia, Philadelphia, Pennsylvania, United States of America; 2 Department of Pathology and Laboratory Medicine, Perelman School of Medicine, University of Pennsylvania, Philadelphia, Pennsylvania, United States of America; 3 Department of Pathology and Cell Biology, College of Physicians and Surgeons, Columbia University, New York, New York, United States of America; 4 Department of Pathology, Cooper University Hospital, Camden, New Jersey, United States of America; 5 Division of Endocrinology, Children's Hospital of Philadelphia, Philadelphia, Pennsylvania, United States of America; 6 Department of Pediatrics Perelman School of Medicine, University of Pennsylvania. Philadelphia, Pennsylvania, United States of America; Laurentian University, Canada

## Abstract

Proteins involved in mitochondrial metabolic pathways engage in functionally relevant multi-enzyme complexes. We previously described an interaction between short-chain 3-hydroxyacyl-coenzyme A dehydrogenase (SCHAD) and glutamate dehydrogenase (GDH) explaining the clinical phenotype of hyperinsulinism in SCHAD-deficient patients and adding SCHAD to the list of mitochondrial proteins capable of forming functional, multi-pathway complexes. In this work, we provide evidence of SCHAD's involvement in additional interactions forming tissue-specific metabolic super complexes involving both membrane-associated and matrix-dwelling enzymes and spanning multiple metabolic pathways. As an example, in murine liver, we find SCHAD interaction with aspartate transaminase (AST) and GDH from amino acid metabolic pathways, carbamoyl phosphate synthase I (CPS-1) from ureagenesis, other fatty acid oxidation and ketogenesis enzymes and fructose-bisphosphate aldolase, an extra-mitochondrial enzyme of the glycolytic pathway. Most of the interactions appear to be independent of SCHAD's role in the penultimate step of fatty acid oxidation suggesting an organizational, structural or non-enzymatic role for the SCHAD protein.

## Introduction

Mitochondrial metabolic pathways provide critical mechanisms for the generation of ATP through oxidative phosphorylation, the tricarboxylic acid cycle and fatty acid beta-oxidation, as well as for the disposal of ammonium through ureagenesis. Previous studies have identified functionally relevant protein-protein interactions across these mitochondrial metabolic pathways. For example, mitochondrial aspartate aminotransferase (AST) has been shown to form complexes with both glutamate dehydrogenase (GDH) in the glutamine degradation pathway and carbamoyl phosphate synthase-1 (CPS-1), the first enzyme committed to ammonium removal. Several groups have independently identified this interaction of GDH with CPS-1 [1]–[3]. Additionally, studies have shown that these physical interactions effect regulation of the individual enzyme activities. Fahien and coworkers demonstrated that CPS-1, and its cofactors ATP and Mg^++^ interact synergistically to facilitate GDH activity and to facilitate the interaction between GDH and AST [4]. This group proposed that these enzymes react in sequence as a multienzyme cluster within the mitochondrial matrix. There is also evidence for direct interaction between individual components of the fatty acid oxidation pathway and those of the respiratory chain [5]–[7].

More recently, a large protein complex association between inner mitochondrial membrane-associated enzymes of fatty acid oxidation and complexes of the respiratory chain has been identified [8]. The physical association between the two energy-generating pathways has long been postulated from overlapping clinical phenotypes in genetic deficiency states [9]–[11] but firm evidence for a complex formation was previously lacking. The studies of Wang et al. [8] also demonstrated association between some mitochondrial matrix enzymes and the fatty acid-respiratory chain complex, including association with isovaleryl-CoA dehydrogenase and short-chain acyl-CoA dehydrogenase. This work provided the first evidence implicating association between membrane-associated complexes and mitochondrial matrix proteins.

In our own studies of the mitochondrial matrix-associated fatty acid oxidation enzyme short-chain 3-hydroxy acyl-CoA dehydrogenase (SCHAD), we have demonstrated a novel regulatory role and distinct physical association between SCHAD and GDH. This interaction defined the mechanism of hyperinsulinism in children with SCHAD deficiency [12]. This study delineated a non-enzymatic role for the SCHAD protein, which, whilst associated with GDH, resulted in down regulation of GDH activity and provided an inhibitory mechanism on insulin release by pancreatic beta cells. In settings in which genetic defects led to an absence of a stable SCHAD protein, this inhibition was lost resulting in unregulated GDH activity and excessive insulin release as described in the patients with mutations that resulted in lack of SCHAD protein [13]–[15]. By contrast, SCHAD- deficient patients who still produced normal sized protein with a physical GDH interaction, such as those with missense mutations in the enzyme, showed no evidence of hyperinsulinism [16].

In earlier pull down studies from murine liver using histidine-tagged SCHAD (His-Tag SCHAD), we recognized that, in addition to GDH, both CPS-1 and 3-hydroxy-3-methylglutaryl-CoA lyase (HMG-CoA lyase) from the hepatic mitochondrial ketogenesis pathway were also identified [12]. To confirm these protein associations with the SCHAD protein and test the hypothesis that there is a multienzyme complex involving several metabolic pathways within the mitochondrial matrix, we performed additional experiments from multiple murine tissues to evaluate the extent and nature of the metabolic complexes present involving SCHAD. Here, we report evidence for large, tissue-specific metabolic complexes involving several important mitochondrial pathways consisting of both matrix-associated proteins and proteins known to be associated with the inner mitochondrial membrane and also, and surprisingly, association of SCHAD with some proteins considered to be extra mitochondrial.

## Materials and Methods

### Mouse Tissues

Mouse tissues from SCHAD knockout and wild type mice were excess tissues that had previously been snap frozen during earlier published experiments [12]. No additional animals were sacrificed for these present experiments.

### Yeast Two Hybrid Analysis

**Figure 1:**
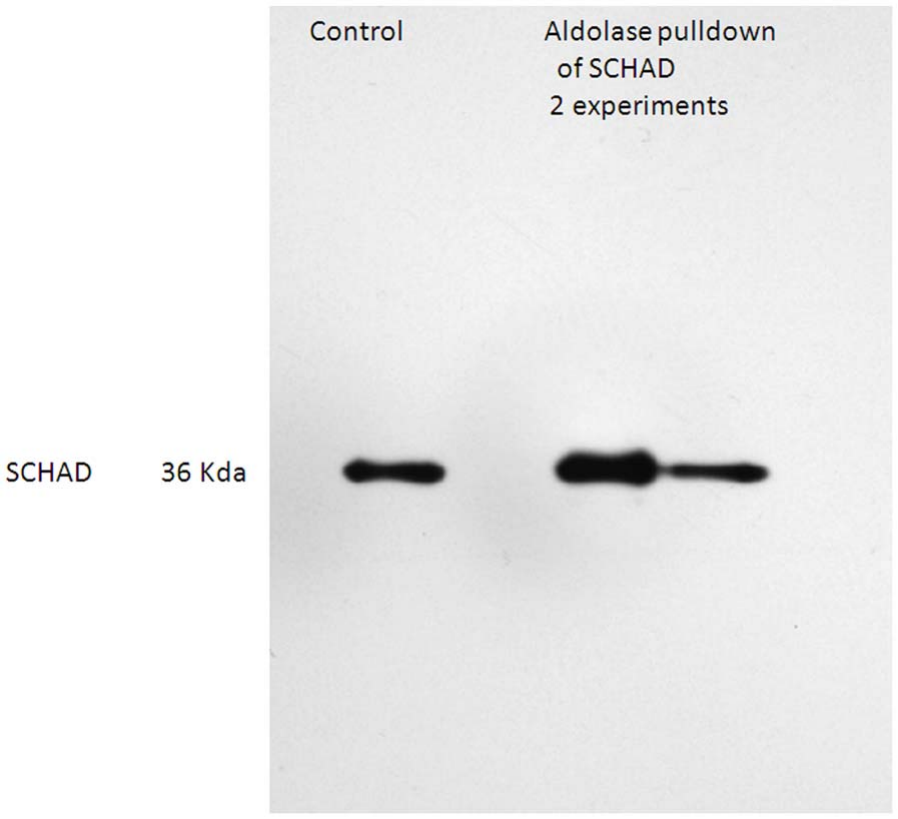
Western blot of SCHAD following pulldown with fructose bisphosphate aldolase. Lane 1, homogenized liver SCHAD control, Lane 2 albumin blank, Lanes 3 and 4 results from 2 separate pulldown experiments showing a normal sized mitochondrial SCHAD.

The DupLEX-A yeast two-hybrid system (OriGene Technologies, Rockville, MD) was used to screen a human placental LexAcDNA library (OriGene Technologies, Rockville, MD) with human SCHAD cDNA encoding amino acids 75–119 fused to LexA in plasmid pEG202 as bait. Fragments of this clone, similarly fused in pEG202, are shown in Fig. 1. Expression of bait proteins was confirmed by Western blot by using LexA antibody. According to the protocol supplied by OriGene, bait plasmid SCHAD 75–119 was tested for its autoactivation potential and ability to enter the nucleus and bind LexA operators. Of ≈1×10^6^ colonies screened, 25 were positive. Plasmids recovered from positive clones were transformed into KC8 bacteria to select colonies containing only target plasmids. The β-galactosidase filter assay was used to assess interaction of SCHAD with other proteins and positives were placed on a grid to small plates, and LacZ filter assay was performed. To confirm the association and identify the site(s) of interaction of SCHAD and positive interactants from initial Lac Z assay, a mating assay was performed. SCHAD and each of the fragments in pEG202 were transformed with pSH18–34 reporter plasmid into RFY206 cells. Proteins coded by cDNA in pJG4.5 (target plasmid) were transformed into EGY48 cells.

### PCR for bait

SCHAD bait was amplified using a human cDNA library prepared from human liver tissue (OriGene Technologies, Rockville, MD). PCR was performed using Amplitaq Gold kit (Applied Biosystems) using the following primers, forward 5′TCCTGGCAAAATCCAAAAAG3′ and reverse 5′ACAGACTTGGTGGTGGAAG3′.

### Protein Pulldown

Recombinant SCHAD (or polyclonal aldolase antibody) linked to agarose beads were used for pulldown experiments from various mouse tissue homogenates using identical conditions described for SCHAD. Recombinant SCHAD was expressed and purified in our laboratory using Ni-his columns (Pierce, Rockford, IL, USA). SCHAD-protein pull down was performed using AminoLinkPlus Immobilization Kit (Pierce, Rockford, IL, USA) following the manufacturer's instructions. Briefly, SCHAD was linked to agarose beads using cyanoborohydride and excess SCHAD protein was washed out. Bovine serum albumin (BSA, fatty acid free, Sigma Chemical, CO, USA) was used as the control protein and immobilized to agarose beads similar to the SCHAD protein.

Tissues were homogenized in 50 mM Tris-HCl buffer containing 0.1% Triton-X 100 and centrifuged at 3000 rpm to separate nuclear and non disrupted tissues. Protein concentration was determined using Lowry's method and protein concentration was adjusted to 1 mg/mL. Homogenates from tissues were incubated for interactions with immobilized SCHAD, polyclonal fructose bisphosphate aldolase antibody or BSA at 4°C overnight. Proteins were eluted from agarose beads following the manufacturer's instructions and identified using mass spectrometric analysis for the SCHAD pulldown experiments and by Western blot using methods we have previously validated for the identification of SCHAD [16].

### Mass spectral identification and validation

Samples were lysed in SDT (4% SDS, 0.1 M Tris-HCl pH 7.6, 0.1 M DTT) and digested with trypsin using the FASP method essentially as described [17]. After C18 StageTip cleanup, the tryptic digests were loaded on a 20 cm×75 um C-18 column packed with 2.7 um Halo beads (Michrom Bioresources). Nanoflow chromatography (300 nL/min) was performed at 60°C (Phoenix S&T column header) using a 90-minute gradient of 2%–>42% acetonitrile in 0.1% formic acid. Online chromatography was coupled to a Thermo Scientific LTQ Orbitrap using a Phoenix S&T AutoNano spray source. A top-5 data-dependent MS/MS method was used with MS acquisition in the Orbitrap and MS/MS fragmentation and measurement in the linear ion trap. All samples were analyzed with two technical replicates. Resulting data files were processed using MaxQuant 1.0.13.13 [18], and spectra were searched with Mascot against the IPI mouse database v. 3.52 with carbamidomethylation of cysteine as a fixed modification and ox (M), N-term acetylation as dynamic modifications. All data were filtered at a 1% FDR (false discovery rate) using a decoy database and a minimum of two peptides were required to confirm the presence of a protein.

### Plasma amino acid analysis

Amino acids were measured in plasma from wild type and knockout mice in both fasted and fed conditions by ultra-performance liquid chromatography using an assay that has been validated for clinical activities [19].

## Results

### Yeast-2 hybrid study

To identify additional protein-protein interactions with SCHAD, we first undertook a yeast 2-hybrid screen using a human SCHAD (AA 77–119)-LexA fusion protein as bait. This portion of SCHAD is shown by X-ray crystallography to form a loop structure that may interact with other proteins. After screening a 2-hybrid library derived from human placental cDNA library, this screen yielded 25 positives clones which encoded 19 different proteins. Sequences were searched against the Genbank database, and SCHAD-interacting proteins identified in these experiments (along with their Genbank accession IDs) are listed in Table 1. Notably, the assay identified several mitochondrial proteins of interest including GDH, AST, and CPS- I. Six of the positive clones encoded tumor necrosis factor receptor-associated factor (TRAF) family proteins.

**Table 1:** Proteins associated with SCHAD using Yeast-2 Hybrid analysis.

Protein	Accession number	Organelle
2-oxoglutarate dehydrogenase E1 component	NM_001003941	Mitochondria
Aldehyde dehydrogenase 3-B1	NM_000694	Mitochondria
ATP synthase subunit A	NM_001001937	Mitochondria
Carbamoyl phosphate synthase 1	NM_001875	Mitochondria
Citrate synthase	NM_004077	Mitochondria
Electron transfer flavoprotein-ubiquinone oxidoreductase	NM_004453	Mitochondria
Glutamate dehydrogenase 1	NM_005271	Mitochondria
3-Hydroxy-3-methyl glutaryl-CoA lyase	NM_000191	Mitochondria
3-hydroxy-3-methyl glutaryl-CoA synthase	NM_005518	Mitochondria
Pyruvate dehydrogenase E1 component subunit alpha	NM_000284	Mitochondria
Pyruvate dehydrogenase E1 component subunit beta	NM_000925	Mitochondria
Pyruvate dehydrogenase protein X component	NM_003477	Mitochondria
Mitochondrial trifunctional protein subunit alpha	NM_000182	Mitochondria
Mitochondrial trifunctional protein subunit beta	NM_0001983	Mitochondria
Fructose-bisphosphate aldolase C	NM_001127617	Cytosol
Glutamine symthase	NM_001033056	Cytosol
Malate dehydrogenase	NM_001199111	Cytosol
Glycogen phosphorylase	NM_002863	Cytosol
TRAF3 interacting protein 2	NM_001164282	Cytosol
TRAF5 interacting protein	NM_001033910	Cytosol
TRA6 interacting protein	NM_004620	Cytosol

### Protein Pulldown

In order to provide a second, independent approach to identify protein-protein interactions of SCHAD, we undertook a pulldown strategy in conjunction with protein identification by tandem mass spectrometry. Histidine-tagged SCHAD was immobilized on agarose beads, and interacting proteins from murine tissue extracts were isolated using affinity chromatography in preparation for LC-MS/MS identification. This approach allowed us to identify conserved SCHAD interactions as well as to extend our initial work across a variety of tissue types. In this study we analyzed tissues from both wild-type and SCHAD knockout mice. This was based on our earlier observation that, at least in pancreas, in the wild type mouse, GDH was fully occupied by SCHAD due to molar excess of SCHAD in this tissue. We determined that there was a risk that proteins such as GDH may not be available to pulldown in the wild type situation. Our results demonstrated that the two murine lines demonstrated comparable pull down profiles across most of the associations. This complementary strategy recognized many of the same protein interactions that we had identified using the yeast-2-hybrid assay thus providing additional evidence that the initial proteins were true interactants with SCHAD. Table 2 provides an abbreviated listing of many of the important associations in different tissues. Supplementary Tables S1,S2,S3,S4,S5 provides a full listing of all proteins identified in association with SCHAD and the number of confirmatory peptides for each protein.) Using the affinity chromatography and proteomics approach, we also identified several other mitochondrial proteins that were not identified using the yeast-2-hybrid approach. The majority of the proteins identified following protein pulldown were enzymes involved in cellular energy metabolism and maintained predicted tissue specificity patterns. This list of interacting proteins includes key regulatory enzymes in metabolic pathways such as fatty acid oxidation, ketogenesis, amino acid catabolism and ureagenesis. Interestingly, we also identified association of SCHAD with some of the enzymes of glycolysis, an extra-mitochondrial energy pathway.

**Table 2:** Proteins found in association with SCHAD by pulldown*.

Pathway	Liver	Muscle	Heart	Kidney	Brain
**Fatty Acid Oxidation**					
carnitine palmitoyl transferase 2	**Yes**	**No**	**No**	**Yes**	**Yes**
very long chain acyl-CoA dehydrogenase	**Yes** **	**No**	**Yes**	**No**	**Yes** **
trifunctional enzyme alpha	**Yes** **	**No**	**Yes**	**No**	**Yes** **
trifunctional enzyme beta	**Yes** **	**No**	**Yes**	**No**	**Yes** **
medium chain acylCoA dehydrogenase	**Yes**	**yes**	**Yes**	**No**	**Yes**
long chain acyl-CoA dehydrogenase	**Yes**	**No**	**Yes**	**No**	**Yes**
3-ketoacyl-coA thiolase	**No**	**No**	**Yes**	**No**	**No**
ACAD 10	**Yes**	**No**	**No**	**No**	**Yes**
**Glutamate/amino acid/urea cycle**					
glutamate dehydrogenase	**Yes**	**No**	**No**	**Yes**	**Yes**
carbamoyl phosphate synthase 1	**Yes**	**No**	**No**	**No**	**No**
argininosuccinate synthase	**Yes**	**No**	**No**	**Yes**	**No**
argininosuccinate lyase	**Yes**	**No**	**No**	**Yes**	**No**
glutamine synthase	**Yes**	**Yes**	**Yes**	**Yes**	**Yes**
aspartate aminotransferase	**Yes**	**Yes**	**No**	**Yes**	**Yes**
**Ketogenesis/ketone utilization**					
succinyl-CoA: 3-ketoacid acyl transferase	**Yes**	**Yes**	**No**	**Yes**	**Yes**
hydroxymethyl-CoA synthase	**Yes**	**No**	**No**	**No**	**No**
hydroxymethyl-coA lyase	**Yes**	**No**	**No**	**No**	**No**
**Oxidative phosphorylation/TCA cycle**					
NADH dehydrogenase flavoprotein 2	**Yes**	**Yes**	**No**	**Yes**	**Yes**
NADH dehydrogenase iron-sulfurprotein2	**Yes**	**No**	**No**	**No**	**No**
**Glycolysis**					
fructose bis phosphate aldolase	**Yes**	**Yes**	**Yes**	**Yes**	**Yes**

* The full proteomic data including numbers of confirming peptides in both wild type and SCHAD knockout mice is provided in the supporting material.

** These long-chain inner mitochondrial-associated enzymes were only seen in the wild-type mouse in liver and brain.

In order to confirm this unexpected association of SCHAD with non-mitochondrial cytosolic enzymes, we undertook additional immunoprecipitation experiments directed at confirming the association with one of the glycolytic enzymes, fructose-bisphosphate aldolase. Pulldown from tissue extracts derived from mice with wild-type SCHAD were undertaken using an anti-aldolase antibody, and Western blot analysis confirmed the copurification of aldolase and SCHAD (Figure 1). Moreover, this analysis demonstrated that the SCHAD species pulled down by fructose bisphosphate aldolase was of equivalent molecular size to the mitochondrial form of SCHAD following intramitochondrial cleavage of the mitochondrial targeting leader peptide. Additional Western blot experimentation using washed red blood cells, which should not express mitochondrial proteins, did not demonstrate the presence of detectable SCHAD protein providing additional support for co-compartmentalization with aldolase.

Our results provide evidence that SCHAD interacts with enzymes from a diverse variety of metabolic pathways and that these enzymes form an interacting metabolic super-complex. The results of our SCHAD pulldown experiments are organized by individual metabolic pathways as well as by tissue specificity.

### Carbohydrate metabolism

The SCHAD pulldown experiments showed that SCHAD protein associated in all tissues with components of the glycolytic pathway and, to a lesser extent, enzymes of the citric acid cycle. The major glycolytic enzymes indentified were fructose bisphosphate aldolase and phosphoglycerate kinase1, and citric acid cycle enzymes included fumarate hydratase and isocitrate dehydrogenase. There was also a consistent association with the proteins of the pyruvate dehydrogenase complex responsible for the generation of acetyl-CoA to fuel the citric acid cycle. We also identified a physical interaction of SCHAD with glycogen phosphorylase, a non-mitochondrial enzyme that is important for the regulation of blood glucose levels from glycogen.

### Glutamate metabolism

SCHAD-interacting proteins that are involved in amino acid metabolism include GDH, AST, responsible for the transamination of glutamate to α-ketoglutarate and glutamine synthetase which converts glutamate to glutamine and also salvages ammonium ions. These proteins were found to be associated with SCHAD in liver, kidney, and brain. We did not identify GDH as an associated protein in skeletal and cardiac muscle, and it is well established that GDH enzyme activity is not expressed in these tissues.

### Lipid metabolism

SCHAD interacted with a variety of membrane-associated and matrix proteins involved in fatty acid β-oxidation in all tissues types tested. Proteins identified include both subunits of the inner mitochondrial membrane-associated trifunctional protein, which contains the 3-hydroxyacyl-CoA dehydrogenase responsible for oxidation of long chain acyl-CoA species; three chain-length specific members of the acyl CoA dehydrogenase family (very long chain acyl-CoA dehydrogenase, medium-chain acyl-CoA dehydrogenase and short-chain acyl-coA dehydrogenase); and 3-keto-acyl CoA thiolase, which is responsible for the subsequent and final reaction of fatty acid oxidation after SCHAD.ACAD10, a recently described member of the acyl-CoA dehydrogenase family is known to be primarily expressed in brain and liver and was only seen in these tissues in our studies. Of particular interest, although present in the wild-type tissue pulldown in liver, heart and brain, we did not see association of SCHAD with the membrane associated enzymes responsible for long-chain fatty acid oxidation in the liver and brain from SCHAD knockout tissues. Cardiac muscle, however, did demonstrate the association in the knock-out mouse.

### Urea cycle

In liver, we identified interactions with CPS-1, argininosuccinate (ASA) synthase and ASA lyase, important components of ureagenesis. As seen with carbohydrate metabolism, in addition to mitochondrial proteins, we identified some enzymes that are localized to the cytosolic fraction (ASA synthase and ASA lyase). We did identify individual enzymes of the urea cycle in other tissues with isolated expression of CPS-1, ASA synthase ASA lyase in association with SCHAD in kidney, and arginase-2, the mitochondrial form of arginase in brain.

### Other pathways

HMG-CoA synthase and HMG-CoA lyase play a role in mitochondrial ketogenesis and were identified in liver using both yeast-2 hybrid and the affinity pulldown-proteomic techniques. Succinyl-CoA: 3-ketoacid acyl transferase, an important enzyme for ketone body utilization was identified in all tissues except the heart muscle. One electron transfer protein (ETF-Ubiquinone oxidoreductase) was identified in liver using the yeast-2 hybrid assay, whilst both ETF and ETF ubiquinone oxidoreductase were identified using pulldown-proteomic techniques in all of the tissues studied. Using the yeast 2 hydbrid technique, we identified three related TNF receptor associated factor family of proteins: TRAF3, 5 and 6. We also identified association of SCHAD with several oxidative phophorylation enzymes in our study, with the majority of them identified in brain. These enzymes include NADH-cytochrome oxidoreductase, cytochrome bc1 complex, succinate dehydrogenase, cytochrome oxidase, and members of the ATP synthase complex.

### Amino acid profiles

Amino acid profiles and blood ammonia levels were obtained from wild type and SCHAD knockout mice in both fasted and fed conditions. A total of 5 animals in each subgroup were studied. We did not detect significant changes in any circulating amino acid level between the wild type or knockout in either fasting or fed status. In particular we did not see evidence of altered ureagenesis through changes in the levels of ammonia, glutamine, ornithine, citrulline or arginine, or alterations in the levels of glutamate or aspartate.

## Discussion

In this study, we have identified protein-protein interactions between SCHAD, the penultimate enzyme of mitochondrial fatty acid β-oxidation and multiple other mitochondrial and some non-mitochondrial metabolic proteins. We initially examined SCHAD interactions using a yeast-2 hybrid system and identified 21 interacting proteins as candidates for a metabolic super-complex. Next, we used immobilized SCHAD on agarose beads coupled with mass spectrometric proteomic analysis to confirm our initial yeast-2-hybrid findings. The process of pulldown with mass-spectrometric identification has greater inherent sensitivity for detecting proteins and has the advantage of allowing us to rapidly test whether different interaction sets are identified within different tissues. Our results from these affinity chromatography protein pull down experiments coupled with mass-spectrometric identification provided valuable verification of the proteins that were identified using the yeast-2 hybrid technique. Using these complimentary approaches, we have confirmed our previously published protein-protein interaction between SCHAD and several other mitochondrial proteins in liver including GDH, CPS1 and HMG-CoA lyase; further, we have revealed new interactions that have not previously been recognized and that together provide evidence for the possible existence of a cellular “metabolic super complex” integrating multiple metabolic pathways.

The identification of these novel interactions underscore a role for the SCHAD protein as an essential component not only of metabolic pathways within the mitochondria but also with those that are not currently regarded as being mitochondrial. Given the surprising nature of this result, we performed reciprocal co-immunoprecipitation to confirm an interaction between SCHAD and fructose bisphosphate aldolase. These experiments appear to confirm a direct or indirect physical interaction between SCHAD and a glycolytic enzyme known to be localized in another intracellular compartment.

Our previous observations of the role of SCHAD in non-enzymatically regulating the function of GDH suggest the possibility that SCHAD might regulate or modulate the function of other critical enzymes through protein-protein interactions [12]. Hardy et al., using a functional genomics approach, observed that SCHAD promotes basal insulin secretion independent of K_ATP_ channels [20]. Several similar studies in which SCHAD was knocked down using shRNA or RNAi techniques in INS832/13 β-cells showed increased glucose stimulated secretion of insulin [21], [22].The increase in the insulin secretion in response to various metabolites such as L-carnitine and 3-hydroxybutyrate was also affected [22]. In our earlier study we were able to demonstrate that GDH activity was up regulated in the absence of SCHAD in a fashion similar to that seen in hyperinsulinism due to gain-of-function mutations in GDH [23], and this provides a paradigm that may be relevant across additional SCHAD protein-protein interactions. Kapoor et al. described novel SCHAD mutations in a patient with severe dietary protein intolerance [24]. We have analyzed plasma amino acids in the SCHAD-deficient mouse to see if the interactions with proteins that are responsible for amino acid and urea cycle pathways provide clues to other regulatory roles of SCHAD in associated pathways or to the protein sensitivity. We could not find any obvious changes in pathways involving ammonia removal through the measurement of urea cycle intermediates or glutamine or to known post-prandial elevations including leucine and the other branched-chain amino acids. It remains possible that SCHAD does not have a direct regulatory role in this pathway but that other components of the complex do. Unlike patients with gain of function mutations in the GDH gene, who demonstrate profound hyperammonemia in association with hyperinsulinemic hypoglycemia [23], patients and the knock out mouse with SCHAD deficiency do not demonstrate hyperammonemia. This suggests that the hyperammonemia seen with GDH mutations may result from an adverse interaction with other protein(s) within the complex.

In our earlier studies on SCHAD-GDH interaction in pancreatic islet cells we observed that the association between these two proteins in the wild-type mouse was complete and that in this scenario GDH was blocked. We were only able to confirm pancreatic islet association in the SCHAD knockout mouse. Subsequently, we performed the current study using tissues from both wild type and SCHAD knockout mice for comparison. The majority of proteins identified by mass spectrometry had similar profiles and recoveries, based on peptide sequence numbers, from both wild type and knockout mice. The major exception that we observed was that in the knock out mouse a number of enzymes of mitochondrial fatty acid oxidation were no longer seen to be associated in some tissues. This was particularly apparent for the trifunctional enzyme subunit beta and very long-chain specific acyl-CoA dehydrogenase in brain and liver and also short-chain specific acyl-CoA dehydrogenase in liver and medium-chain specific acyl-CoA dehydrogenase in skeletal muscle. However, all of these enzymes were identified as equal components in both wild type and SCHAD knockout cardiac muscle. Taken together, this evidence suggests that the loss of SCHAD does result in loss of both membrane associated and matrix associated members of the fatty acid oxidation pathway in some tissues due to structural changes in the super-complex. This loss of structure is not seen in cardiac muscle suggesting a more stable complex in heart which is a tissue that has an obligate need for fatty acid oxidation as an energy source. Also, cardiac muscle was the only tissue that did not show association with succinyl-CoA: 3-ketoacid acyl transferase, an important enzyme in ketone body utilization as an energy source, indicating that ketones may not be important fuels for heart energetics.

We also identified associations between SCHAD and several urea cycle enzymes, including the extra-mitochondrial ASA synthase and ASA lyase in liver. We speculate that the functional significance of the association between the SCHAD and urea cycle enzymes may be related to the requirement of N-acetylglutamate (NAG) as an activator of the first enzymatic step of ureagenesis, which is catalyzed by CPS-1. The end product of fatty acid oxidation through the thiolase step is acetyl-CoA which in addition to glutamate is essential for activation of CPS-1. Hardy et al. [20] demonstrated that inhibition of ASA synthase 1 expression by RNAi techniques resulted in decreased secretion of insulin. This suggests that ASA synthase is a potential activator of insulin secretion, perhaps through its interaction with the known down-regulator of GDH activity, SCHAD. This also provides further evidence that is consistent with our proposal of a metabolic super-complex. Of note, the rate-limiting enzyme in ureagenesis, CPS-1, was associated with SCHAD only in the liver. Expression of CPS-1 is limited to the liver and kidney and the expectation is that interaction should only occur in a tissue-specific manner. Although more studies are necessary to confirm the physiological relevance of these associations, it is clear that SCHAD has a potential role as a modulator in the urea cycle. Additionally, in liver (but not other tissues) we identified an association with the two enzymes of ketone body formation, HMG-CoA synthase and HMG-CoA lyase. We would not expect to see these proteins in other tissues where ketone bodies are not synthesized.

Our pull down studies revealed an interaction between SCHAD and several components components of the mitochondrial oxidative phosphorylation system. Interactions with several functional components of the OX/PHOS system, including NADH-ubiquinone oxidoreductase, succinate dehydrogenase, cytochrome bc1 complex, cytochrome c oxidase and others, were identified. In contrast, the relative lack of OX/PHOS structural components identified suggests that interactions of SCHAD may be more specific to catalytic components of this pathway. This is in keeping with the role of SCHAD and also the trifunctional protein as NADH-generating enzymes demonstrating a logical physical association with the regeneration of NAD through the respiratory chain.

Our yeast-2-hybrid studies have identified physical interactions between SCHAD and some of the TRAF family of proteins, specifically TRAF3, 5 and 6. Studies of the TRAF6 knockout mouse model have shown a defect in mitochondrial fatty acid oxidation as well as in AMP activated kinase activation in CD8^+^ T cells [25]. These results suggest that TRAF family proteins may play important regulatory roles in intermediary metabolic pathways both within and outside of the mitochondrion.

**Figure 2:**
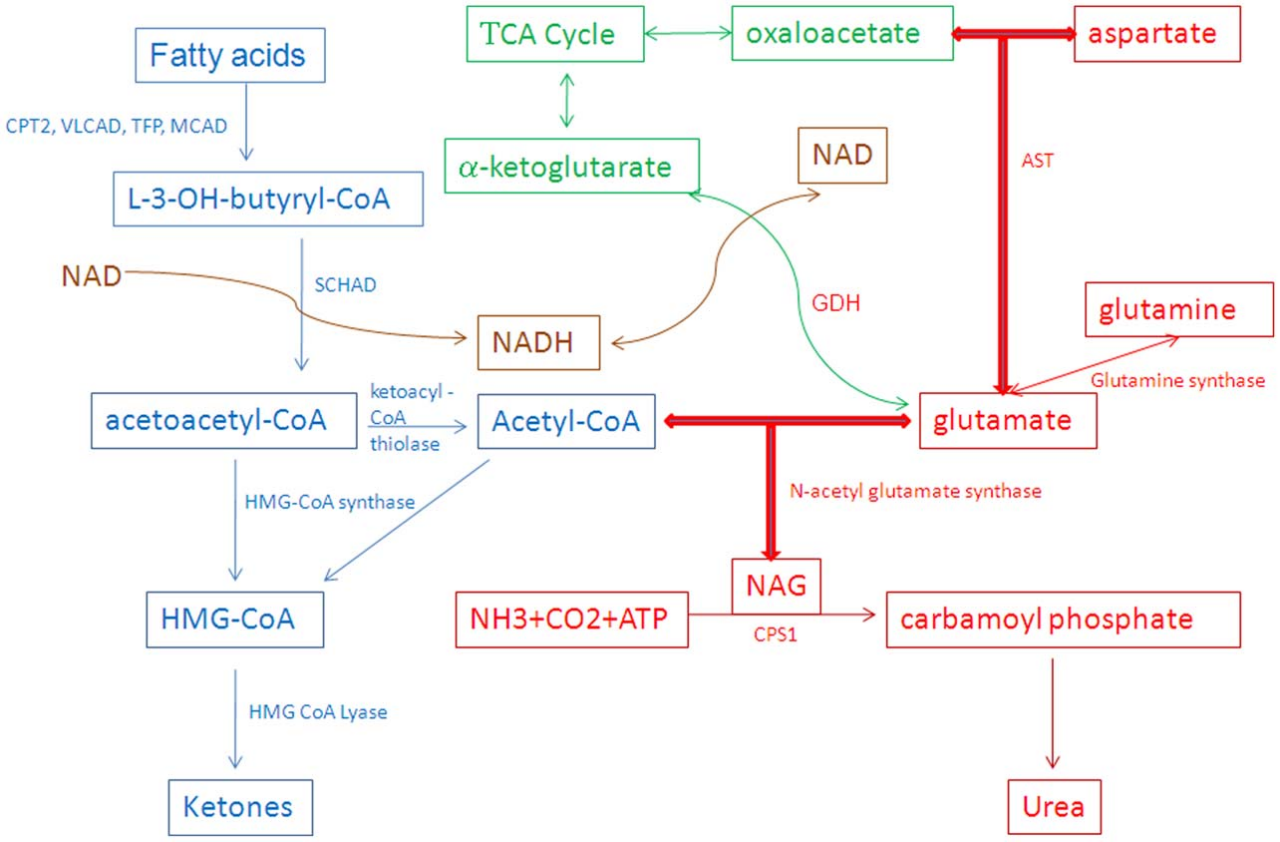
Interactions of several metabolic pathways represented as components of the proposed mitochondrial matrix super complex. The pathways include fatty acid oxidation, ketogenesis, glutamate transamination, ureagenesis and the TCA cycle.

A model of how SCHAD and its associated proteins link the metabolic pathways in the mitochondria is shown in Figure 2. Several of the pathways that have been shown to associate are centered on glutamate metabolism. The primary function of SCHAD is the conversion of medium and short-chain L-3-hydroxyacyl–CoA's to their respective 3-ketoacyl-CoA's. Based on the pathway components that our study identified results we have depicted SCHAD interactions with enzymatic mediators of ureagenesis, ketogenesis, oxidative phosphorylation, the citric acid cycle and transamination in addition to its known enzymatic role in fatty acid oxidation. HMG CoA synthase, CPS1, and GDH are regulatory components of their respective pathways. Wang et al., [8] demonstrated the association of mitochondrial fatty acid oxidation enzymes and oxidative phosphorylation complexes using blue native gel electrophoresis techniques. This was the first study that established a metabolic complex between mitochondrial membrane and matrix protein associations. In our present study, we have identified a similar complex of both matrix and membrane-associated proteins using SCHAD, which is a mitochondrial matrix protein.

Hardy et al, [20] utilized a functional genomics approach to look at possible interactions of SCHAD and identified several of the proteins that we report. Our previous studies and those of others provide support by other studies from and provide support that the physical interactions of SCHAD, separate and apart from its primary enzymatic role, may be important in modulating multiple pathways of metabolism [8], [20], [22].

In conclusion, we have demonstrated a physical association between SCHAD and important components of other key metabolic pathways. Most of the interactions are with enzymes in mitochondrial pathways and most of the interactions appear to be independent of the primary role of SCHAD as an enzyme responsible for the penultimate step of fatty acid oxidation. These interactions would define a metabolic super-complex where multiple metabolic pathways converge. Unexpectedly, we also identified a number of non-mitochondrial protein reactions on classical pathways that are related to those of the mitochondria including energy metabolism through glycolysis and ureagenesis through extra-mitochondrial urea cycle proteins. Pulldown using fructose bisphosphate aldolase confirmed these associations. It is well known that glycolytic enzymes associate with mitochondria [26] but direct associations so far have been limited to membrane constituents including H^+^ ATPases suggesting a role in regulating mitochondrial membrane potential [27]. A recent study by Genda and coworkers identified co-compartmentalization of an astroglial glutamate transporter with glycolytic enzymes and mitochondria using methods that are similar to ours and providing additional evidence to support the association of glycolysis and mitochondrial metabolism [28]. Association of glycolytic enzymes with mitochondrial matrix proteins has not previously been described but we consider that our observation that this is so using pulldown with both proteins suggests that this is a real phenomenon. We were unable to demonstrate the presence of SCHAD protein outside of the mitochondria as demonstrated by our western blot analysis of red blood cell preparations. A possible linkage mechanism between the matrix proteins and those of glycolysis may come through the mitochondrial membrane associated proteins that we detected in our association studies. As we consider it to be highly unlikely that the enzymatic activity of SCHAD is required as part of these observed interactions, we suggest additional structural and potential regulatory roles for SCHAD. Potential regulatory roles between the associated proteins form the basis of our ongoing studies and the full nature of this association remains to be elucidated.

## Supporting Information

Table S1 **Full pulldown proteomic details from wild type and SCHAD knockout brain.**
(DOCX)

Table S2 **Full pulldown proteomic details from wild type and SCHAD knockout kidney.**
(DOCX)

Table S3 **Full pulldown proteomic details from wild type and SCHAD knockout heart.**
(DOCX)

Table S4 **Full pulldown proteomic details from wild type and SCHAD knockout skeletal muscle.**
(DOCX)

Table S5 **Full pulldown proteomic details from wild type and SCHAD knockout liver.**
(DOCX)
