# Supplementary material for: Short-Chain 3-Hydroxyacyl-Coenzyme A Dehydrogenase Associates with a Protein Super-Complex Integrating Multiple Metabolic Pathways
Source: PLoS One. 2012 Apr 9;7(4):e35048. doi: 10.1371/journal.pone.0035048 (PMC3322157; doi:10.1371/journal.pone.0035048)
Supplement: Table S1 — Full pulldown proteomic details from wild type and SCHAD knockout brain. (DOCX) [file pone.0035048.s001.docx]

| **Supplementary Table S1 Proteins identified and number of confirmatory peptides in SCHAD pulldown experiments from wild type and SCHAD knockout mouse brain** | **WT** | **KO** |
| --- | --- | --- |
| **Glycolysis** |  |  |
| 78 kDa glucose-regulated protein;GRP 78;Heat shock 70 kDa protein 5;Immunoglobulin heavy chain-binding protein | 14 | 19 |
| Pyruvate dehydrogenase E1 component subunit alpha | 29 | 21 |
| Pyruvate dehydrogenase E1 component subunit beta | 28 | 18 |
| Pyruvate dehydrogenase protein X component | 11 | 12 |
| Fructose-bisphosphate aldolase B;Liver-type aldolase;Aldolase 2 | 10 | 10 |
| Fructose-bisphosphate aldolase C | 13 | 16 |
| Fructose-bisphosphate aldolase;Fructose-bisphosphate aldolase A | 16 | 18 |
| Gamma-enolase | 7 | 21 |
| Aldehyde dehydrogenase | 20 | 18 |
| **TCA Cycle** |  |  |
| Fumarate hydratase | 24 | 16 |
| Dihydrolipoyl dehydrogenase | 16 | 13 |
| Dihydrolipoyllysine-residue succinyltransferase component of 2-oxoglutarate dehydrogenase complex | 12 | 15 |
| Isocitrate dehydrogenase [NAD] subunit alpha | 8 | 9 |
| Isocitrate dehydrogenase [NADP] | 24 | 22 |
| **Mitochondrial and Energy metabolism** | |  |
| ATP synthase subunit alpha | 23 | 20 |
| ATP synthase subunit beta | 26 | 24 |
| ATP synthase subunit O | 13 | 9 |
| ATP synthase-coupling factor 6 | 5 | 6 |
| ATPase | 15 | 14 |
| NADH dehydrogenase [ubiquinone] flavoprotein 2 | 11 | 14 |
| NADH dehydrogenase [ubiquinone] iron-sulfur protein 2 | 13 | 13 |
| NADH-ubiquinone oxidoreductase 75 kDa subunit | 47 | 37 |
| V-type proton ATPase catalytic subunit A | 28 | 26 |
| Creatine kinase | 18 | 15 |
| Creatine kinase B-type;Creatine kinase B chain;B-CK | 29 | 20 |
| Creatine kinase M-type;Creatine kinase M chain;M-CK | 35 | 26 |
| Cytochrome b-c1 complex subunit 1 | 24 | 19 |
| Cytochrome b-c1 complex subunit 2 | 10 | 21 |
| Cytochrome b-c1 complex subunit Rieske | 10 | 10 |
| Cytochrome c oxidase | 8 | 10 |
| NADH dehydrogenase [ubiquinone] flavoprotein 1 | 20 | 19 |
| NADH dehydrogenase [ubiquinone] flavoprotein 2 | 11 | 14 |
| NADH dehydrogenase [ubiquinone] iron-sulfur protein 2 | 13 | 13 |
| V-type proton ATPase subunit C 1;Vacuolar proton pump subunit C 1 | 9 | 9 |
| V-type proton ATPase subunit d 1;Vacuolar proton pump subunit d 1 | 11 | 11 |
| V-type proton ATPase subunit D | 11 | 10 |
| V-type proton ATPase subunit E 1 | 10 | 13 |
| V-type proton ATPase subunit F | 8 | 10 |
| L-lactate dehydrogenase B chain;LDH heart subunit | 22 | 17 |
| L-lactate dehydrogenase | 29 | 22 |
| **Amino Acids** |  |  |
| Glutamate dehydrogenase 1 | 21 | 20 |
| Aspartate aminotransferase | 17 | 19 |
| Glutamine synthetase;Glutamate--ammonia ligase | 16 | 11 |
| Glutathione S-transferase Mu 1;GST class-mu 1;Glutathione S-transferase GT8.7;pmGT10;GST 1-1;Glutathione S-transferase | 22 | 16 |
| Inorganic pyrophosphatase 2 | 15 | 16 |
| Glycine amidinotransferase | 18 | 15 |
| Ornithine aminotransferase | 16 | 15 |
| **Fatty Acid Oxidation** |  |  |
| Acyl-coenzyme A synthetase ACSM2 | 15 | 15 |
| Trifunctional enzyme subunit alpha | 23 | 25 |
| Trifunctional enzyme subunit beta | 23 | 4 |
| Very long-chain specific acyl-CoA dehydrogenase | 31 | 2 |
| Carnitine palmitoyltransferase 2 | 8 | 8 |
| Hydroxyacyl-coenzyme A dehydrogenase | 11 | 1 |
| Long-chain specific acyl-CoA dehydrogenase | 17 | 19 |
| Acyl-Coenzyme A oxidase 1 | 5 | 5 |
| Medium-chain specific acyl-CoA dehydrogenase | 21 | 19 |
| Acyl-CoA dehydrogenase family member 10 | 11 | 12 |
|  |  |  |
| Acetyl-CoA acetyltransferase | 12 | 13 |
| Acetyl-coenzyme A synthetase 2-like | 12 | 12 |
| Acyl-coenzyme A synthetase ACSM2 | 15 | 15 |
| Enoyl-CoA hydratase | 11 | 10 |
| **Urea Cycle** |  |  |
| Arginase-2 | 7 | 7 |
| **Others** |  |  |
| Inorganic pyrophosphatase 2 | 15 | 16 |
| Citrate synthase | 15 | 12 |
| Glycogen phosphorylase | 35 | 49 |
| Succinyl-CoA:3-ketoacid-coenzyme A transferase 1 | 8 | 8 |
| Dihydrolipoamide branched chain transacylase E2 | 22 | 20 |
| 2-oxoglutarate dehydrogenase E1 component | 24 | 24 |
| 3-hydroxyisobutyrate dehydrogenase | 11 | 12 |
| 4-trimethylaminobutyraldehyde dehydrogenase | 10 | 10 |
| Homogentisate 1 | 18 | 16 |
| Malate dehydrogenase | 19 | 16 |
| Methylcrotonoyl-CoA carboxylase beta chain | 17 | 17 |
| Methylcrotonoyl-CoA carboxylase subunit alpha | 12 | 12 |
| Methylmalonate-semialdehyde dehydrogenase [acylating] | 19 | 18 |
| Sulfite oxidase | 11 | 11 |
| Propionyl-CoA carboxylase alpha chain | 20 | 20 |
| Fumarylacetoacetase | 16 | 14 |
