# Supplementary material for: Short-Chain 3-Hydroxyacyl-Coenzyme A Dehydrogenase Associates with a Protein Super-Complex Integrating Multiple Metabolic Pathways
Source: PLoS One. 2012 Apr 9;7(4):e35048. doi: 10.1371/journal.pone.0035048 (PMC3322157; doi:10.1371/journal.pone.0035048)
Supplement: Table S2 — Full pulldown proteomic details from wild type and SCHAD knockout kidney. (DOCX) [file pone.0035048.s002.docx]

| **Supplementary Table S2 Proteins identified and number of confirmatory peptides in SCHAD pulldown experiments from wild type and SCHAD knockout mouse kidney** | **WT** | **KO** |  |
| --- | --- | --- | --- |
| **Glycolysis** |  |  |  |
| 78 kDa glucose-regulated protein | 16 | 20 |  |
| Aconitate hydratase | 20 | 15 |  |
| Beta-enolase;2-phospho-D-glycerate hydro-lyase | 13 | 16 |  |
| Fructose-bisphosphate aldolase B;Liver-type aldolase;Aldolase 2 | 14 | 16 |  |
| Fructose-bisphosphate aldolase | 12 | 12 |  |
| Glyceraldehyde-3-phosphate dehydrogenase | 12 | 10 |  |
| Phosphoglycerate kinase 1 | 13 | 8 |  |
| Phosphoglycerate mutase 1;Phosphoglycerate mutase isozyme B;BPG-dependent PGAM 1 | 5 | 2 |  |
| Phosphoglycerate mutase 2 | 4 | 5 |  |
| **TCA Cycle** |  |  |  |
| Fumarate hydratase | 15 | 12 |  |
| Isocitrate dehydrogenase [NAD] subunit alpha | 5 | 2 |  |
| Isocitrate dehydrogenase [NADP] | 19 | 19 |  |
| Succinate dehydrogenase [ubiquinone] flavoprotein subunit | 22 | 17 |  |
| Succinate dehydrogenase [ubiquinone] iron-sulfur subunit | 7 | 8 |  |
| Succinyl-CoA ligase [GDP-forming] subunit alpha | 3 | 4 |  |
| Succinyl-CoA ligase [GDP-forming] subunit beta | 3 | 1 |  |
| Succinyl-CoA:3-ketoacid-coenzyme A transferase 1 | 5 | 2 |  |
| **Mitochondrial and Energy Metabolism** |  |  |  |
| Dihydrolipoyllysine-residue acetyltransferase component of pyruvate dehydrogenase complex | 18 | 15 |  |
| Dihydrolipoyllysine-residue succinyltransferase component of 2-oxoglutarate dehydrogenase complex | 9 | 4 |  |
| Electron transfer flavoprotein subunit alpha | 11 | 10 | |
| Electron transfer flavoprotein subunit beta | 10 | 9 |  |
| Electron transfer flavoprotein-ubiquinone oxidoreductase | 15 | 13 |  |
| Pyruvate kinase isozymes M1/M2;Pyruvate kinase muscle isozyme | 9 | 21 |  |
| Cytochrome b-c1 complex subunit 1 | 16 | 15 |  |
| Cytochrome b-c1 complex subunit 2 | 15 | 15 |  |
| Creatine kinase | 4 | 4 |  |
| Creatine kinase B-type;Creatine kinase B chain;B-CK | 11 | 8 |  |
| Creatine kinase M-type;Creatine kinase M chain;M-CK | 18 | 17 |  |
| ATP synthase subunit beta | 40 | 27 |  |
| ATP synthase subunit d | 14 | 12 |  |
| NADH dehydrogenase [ubiquinone] flavoprotein 1 | 15 | 17 |  |
| NADH dehydrogenase [ubiquinone] flavoprotein 2 | 11 | 10 |  |
| **Amino Acid** |  |  |  |
| Glutamate dehydrogenase 1 | 6 | 6 |  |
| Aspartate aminotransferase | 13 | 10 |  |
| Aminoacylase-1;N-acyl-L-amino-acid amidohydrolase;ACY-1 | 12 | 4 |  |
| 2-amino-3-carboxymuconate-6-semialdehyde decarboxylase | 6 | 4 |  |
| L-asparaginase;L-asparagine amidohydrolase;Asparaginase-like protein 1 | 3 | 6 |  |
| Ornithine aminotransferase | 5 | 10 |  |
| Glutathione S-transferase Mu 1 | 10 | 10 |  |
| Glutamine synthetase;Glutamate--ammonia ligase | 5 | 5 |  |
| **Fatty Acid Oxidation** |  |  |  |
| Acetyl-CoA acetyltransferase | 3 | 3 |  |
| Acetyl-CoA acetyltransferase | 3 | 3 |  |
| Acetyl-coenzyme A synthetase 2-like | 2 | 2 |  |
| Carboxylesterase 3;Triacylglycerol hydrolase;Fatty acid ethyl ester synthase | 13 | 4 |  |
| Carnitine palmitoyltransferase 2 | 4 | 1 |  |
| Low-density lipoprotein receptor-related protein 2;Megalin;Glycoprotein 330 | 20 | 33 |  |
| Acetyl-Coenzyme A acyltransferase 2 | 26 | 21 |  |
| Enoyl-CoA hydratase | 7 | 6 |  |
| **Urea Cycle** |  |  |  |
| Argininosuccinate lyase;Arginosuccinase | 11 | 10 |  |
| Argininosuccinate synthase;Citrulline--aspartate ligase | 18 | 3 |  |
| **Others** |  |  |  |
| 60 kDa heat shock protein | 22 | 11 |  |
| Catalase | 12 | 13 |  |
| Cubilin (Intrinsic factor-cobalamin receptor);Cubilin;Intrinsic factor-cobalamin receptor | 12 | 9 |  |
| Delta-aminolevulinic acid dehydratase;Porphobilinogen synthase | 7 | 11 |  |
| Dihydropyrimidinase-related protein 2;ULIP 2 protein | 13 | 12 |  |
| Glutathione peroxidase 1;GSHPx-1;Cellular glutathione peroxidase | 10 | 5 |  |
| Heat shock cognate 71 kDa protein;Heat shock 70 kDa protein 8 | 23 | 20 |  |
| Peroxiredoxin-1;Thioredoxin peroxidase 2 | 10 | 8 |  |
| Peroxiredoxin-2;Thioredoxin peroxidase 1 | 5 | 8 |  |
| Peroxiredoxin-5 | 11 | 9 |  |
| Peroxiredoxin-6;Antioxidant protein 2 | 9 | 7 |  |
| Serotransferrin;Siderophilin;Beta-1 metal-binding globulin | 26 | 16 |  |
| Malate dehydrogenase | 11 | 2 |  |
| Triosephosphate isomerase;Triose-phosphate isomerase | 12 | 11 |  |
| L-lactate dehydrogenase B chain;LDH heart subunit | 15 | 14 |  |
| L-lactate dehydrogenase;L-lactate dehydrogenase A chain;LDH muscle subunit;Ldha protein | 14 | 15 |  |
| Glycogen phosphorylase | 12 | 12 |  |
