# Supplementary material for: Short-Chain 3-Hydroxyacyl-Coenzyme A Dehydrogenase Associates with a Protein Super-Complex Integrating Multiple Metabolic Pathways
Source: PLoS One. 2012 Apr 9;7(4):e35048. doi: 10.1371/journal.pone.0035048 (PMC3322157; doi:10.1371/journal.pone.0035048)
Supplement: Table S3 — Full pulldown proteomic details from wild type and SCHAD knockout heart. (DOCX) [file pone.0035048.s003.docx]

| **Supplementary Table S3 Proteins identified and number of confirmatory peptides in SCHAD pulldown experiments from wild type and SCHAD knockout mouse heart** | **WT** | **KO** |
| --- | --- | --- |
| **Glycolysis** |  |  |
| 78 kDa glucose-regulated protein | 15 | 14 |
| Aldehyde dehydrogenase | 20 | 17 |
| Fructose-bisphosphate aldolase | 22 | 18 |
| Glucose-6-phosphate isomerase | 8 | 11 |
| Glyceraldehyde-3-phosphate dehydrogenase | 14 | 12 |
| Gamma-enolase;2-phospho-D-glycerate hydro-lyase | 16 | 19 |
| Phosphoglycerate kinase 1 | 7 | 10 |
| Phosphoglycerate mutase 1 | 6 | 8 |
| Alpha-enolase;2-phospho-D-glycerate hydro-lyase | 13 | 16 |
| **TCA Cycle** |  |  |
| Pyruvate dehydrogenase E1 component subunit alpha | 29 | 21 |
| Pyruvate dehydrogenase E1 component subunit beta | 28 | 18 |
| Pyruvate kinase isozymes M1/M2;Pyruvate kinase muscle isozyme | 14 | 19 |
| Dihydrolipoyllysine-residue acetyltransferase component of pyruvate dehydrogenase complex | 11 | 11 |
| Dihydrolipoyl dehydrogenase | 16 | 13 |
| Fumarate hydratase | 8 | 10 |
| 2-oxoglutarate dehydrogenase E1 component | 24 | 24 |
| **Mitochondrial and Energy Metabolism** |  |  |
| ATP synthase subunit alpha | 24 | 28 |
| ATP synthase subunit beta | 40 | 27 |
| Creatine kinase | 5 | 4 |
| Creatine kinase B-type;Creatine kinase B chain;B-CK | 16 | 14 |
| V-type proton ATPase catalytic subunit A | 28 | 28 |
| V-type proton ATPase subunit B | 23 | 23 |
| L-lactate dehydrogenase B chain;LDH heart subunit | 7 | 13 |
| L-lactate dehydrogenase;L-lactate dehydrogenase A chain | 10 | 11 |
| **Amino Acid** |  |  |
| Glutamine synthetase;Glutamate--ammonia ligase | 15 | 15 |
| Glutathione S-transferase A4 | 7 | 6 |
| Glutathione S-transferase Mu 1 | 12 | 1 |
| Glutathione S-transferase Mu 2 | 2 | 2 |
| Glutathione S-transferase Mu 5;GST class-mu 5;Fibrous sheath component 2 | 3 | 7 |
| Glutathione S-transferase P 1 | 7 | 7 |
| Glutathione S-transferase theta-2;GST class-theta-2;Glutathione S-transferase | 3 | 3 |
| **Fatty Acid Oxidation** |  |  |
| 3-ketoacyl-CoA thiolase A | 2 | 2 |
| Acetyl-CoA acetyltransferase | 12 | 13 |
| Acyl-Coenzyme A oxidase 1 | 5 | 5 |
| Fatty acid-binding protein | 2 | 7 |
| Medium-chain specific acyl-CoA dehydrogenase | 21 | 19 |
| Trifunctional enzyme subunit alpha | 23 | 25 |
| Trifunctional enzyme subunit beta | 23 | 30 |
| Long-chain specific acyl-CoA dehydrogenase | 17 | 19 |
| Very long-chain specific acyl-CoA dehydrogenase | 31 | 31 |
| **Others** |  |  |
| Heat shock cognate 71 kDa protein;Heat shock 70 kDa protein 8;Putative uncharacterized protein | 21 | 20 |
| Heat shock protein beta-1;Heat shock 27 kDa protein;HSP 27;Growth-related 25 kDa protein;P25;HSP25 | 10 | 12 |
| Heat shock protein beta-2 | 7 | 4 |
| 60 kDa heat shock protein | 11 | 4 |
| Heat shock protein beta-6 | 5 | 5 |
| Heat shock 70 kDa protein 1B | 7 | 3 |
| Heat shock 70 kDa protein 1L | 3 | 2 |
| Methylcrotonoyl-CoA carboxylase subunit alpha | 12 | 12 |
| Isovaleryl-CoA dehydrogenase | 10 | 10 |
| Malate dehydrogenase | 19 | 16 |
| Myosin-4;Myosin heavy chain 4 | 57 | 66 |
| Phosphoribosylaminoimidazole carboxylase | 20 | 23 |
| Propionyl-CoA carboxylase alpha chain | 20 | 20 |
