# Supplementary material for: Short-Chain 3-Hydroxyacyl-Coenzyme A Dehydrogenase Associates with a Protein Super-Complex Integrating Multiple Metabolic Pathways
Source: PLoS One. 2012 Apr 9;7(4):e35048. doi: 10.1371/journal.pone.0035048 (PMC3322157; doi:10.1371/journal.pone.0035048)
Supplement: Table S4 — Full pulldown proteomic details from wild type and SCHAD knockout skeletal muscle. (DOCX) [file pone.0035048.s004.docx]

| **Supplementary Table S4 Proteins identified and number of confirmatory peptides in SCHAD pulldown experiments from wild type and SCHAD knockout mouse skeletal muscle** | **SWT** | **KO** |
| --- | --- | --- |
| **Glycolysis** |  |  |
| 78 kDa glucose-regulated protein | 6 | 1 |
| Fructose-bisphosphate aldolase | 24 | 31 |
| Glucose-6-phosphate isomerase | 11 | 9 |
| Phosphoglycerate kinase 1 | 11 | 12 |
| Phosphoglycerate mutase 1 | 2 | 6 |
| Alcohol dehydrogenase [NADP | 10 | 8 |
| Glyceraldehyde-3-phosphate dehydrogenase | 15 | 13 |
| Pyruvate dehydrogenase E1 component subunit alpha | 18 | 11 |
| Pyruvate dehydrogenase E1 component subunit beta | 18 | 12 |
| Pyruvate dehydrogenase protein X component | 6 | 7 |
| **TCA cycle** |  |  |
| Isocitrate dehydrogenase [NAD] subunit alpha | 4 | 4 |
| Isocitrate dehydrogenase [NADP] | 14 | 8 |
| Aconitate hydratase | 2 | 12 |
| Fumarate hydratase | 12 | 12 |
| Succinate dehydrogenase [ubiquinone] flavoprotein subunit | 9 | 19 |
| Succinate dehydrogenase [ubiquinone] iron-sulfur subunit | 7 | 7 |
| Succinyl-CoA ligase [GDP-forming] subunit beta | 8 | 0 |
| Succinyl-CoA:3-ketoacid-coenzyme A transferase 1 | 6 | 7 |
| Dihydrolipoyl dehydrogenase | 13 | 2 |
| Dihydrolipoyllysine-residue acetyltransferase component of pyruvate dehydrogenase complex | 7 | 15 |
| Dihydrolipoyllysine-residue succinyltransferase component of 2-oxoglutarate dehydrogenase complex | 7 | 8 |
| **Mitochondrial and Energy Metabolism** |  |  |
| Electron transfer flavoprotein subunit alpha | 3 | 7 |
| Electron transfer flavoprotein subunit beta | 2 | 10 |
| Electron transfer flavoprotein-ubiquinone oxidoreductase | 3 | 13 |
| NADH dehydrogenase [ubiquinone] flavoprotein 1 | 12 | 14 |
| NADH dehydrogenase [ubiquinone] flavoprotein 2 | 8 | 10 |
| NADH-ubiquinone oxidoreductase 75 kDa subunit | 34 | 31 |
| Creatine kinase | 9 | 10 |
| Creatine kinase | 4 | 11 |
| Creatine kinase B-type;Creatine kinase B chain;B-CK | 5 | 10 |
| Cytochrome b-c1 complex subunit 1 | 12 | 17 |
| Cytochrome b-c1 complex subunit 2 | 12 | 8 |
| **Amino Acids** |  |  |
| Glutamate dehydrogenase 1 | 1 | 0 |
| Glutamine synthetase;Glutamate--ammonia ligase | 15 | 8 |
| Methylcrotonoyl-CoA carboxylase beta chain | 9 | 14 |
| Aspartate aminotransferase | 10 | 16 |
| Glutathione S-transferase A4 | 4 | 7 |
| Glutathione S-transferase Mu 1 | 9 | 6 |
| Glutathione S-transferase Mu 2 | 8 | 4 |
| Glutathione S-transferase Mu 5;GST class-mu 5;Fibrous sheath component 2 | 7 | 2 |
| Ornithine aminotransferase | 14 | 15 |
| **Fatty Acid Oxidation** |  |  |
| Acetyl-CoA acetyltransferase | 11 | 2 |
| Acetyl-coenzyme A synthetase 2-like | 10 | 2 |
| Enoyl-CoA hydratase | 7 | 6 |
| Fatty acid-binding protein | 2 | 4 |
| Fatty acid-binding protein | 6 | 2 |
| Hydroxyacyl-coenzyme A dehydrogenase | 6 | 2 |
| 3-hydroxyisobutyrate dehydrogenase | 10 | 5 |
| Medium-chain specific acyl-CoA dehydrogenase | 14 | 2 |
| **Others** |  |  |
| Pyruvate kinase isozymes M1/M2;Pyruvate kinase muscle isozyme | 22 | 16 |
| Malate dehydrogenase | 13 | 9 |
| Methylcrotonoyl-CoA carboxylase beta chain | 9 | 14 |
| Carbonic anhydrase 3;Carbonic anhydrase III | 14 | 13 |
| L-lactate dehydrogenase B chain | 15 | 15 |
|  |  |  |
|  |  |  |
|  |  |  |
|  |  |  |
