# Supplementary material for: Short-Chain 3-Hydroxyacyl-Coenzyme A Dehydrogenase Associates with a Protein Super-Complex Integrating Multiple Metabolic Pathways
Source: PLoS One. 2012 Apr 9;7(4):e35048. doi: 10.1371/journal.pone.0035048 (PMC3322157; doi:10.1371/journal.pone.0035048)
Supplement: Table S5 — Full pulldown proteomic details from wild type and SCHAD knockout liver. (DOCX) [file pone.0035048.s005.docx]

| **Supplementary Table S5 Proteins identified and number of confirmatory peptides in SCHAD pulldown experiments from wild type and SCHAD knockout mouse liver** | **WT** | **KO** |
| --- | --- | --- |
| **Glycolysis** |  |  |
| 78 kDa glucose-regulated protein | 16 | 20 |
| Aconitate hydratase | 20 | 15 |
| Aldehyde dehydrogenase | 20 | 18 |
| Beta-enolase;2-phospho-D-glycerate hydro-lyase | 13 | 16 |
| Fructose-bisphosphate aldolase B;Liver-type aldolase;Aldolase 2 | 24 | 26 |
| Fructose-bisphosphate aldolase C | 13 | 16 |
| Fructose-bisphosphate aldolase | 28 | 30 |
| Glycogen phosphorylase | 35 | 49 |
| Phosphoglycerate kinase 1 | 13 | 8 |
| Phosphoglycerate mutase 1;Phosphoglycerate mutase isozyme B;BPG-dependent PGAM 1 | 5 | 2 |
| Phosphoglycerate mutase 2 | 4 | 5 |
| Glyceraldehyde-3-phosphate dehydrogenase | 12 | 10 |
| **TCA Cycle** |  |  |
| Isocitrate dehydrogenase [NAD] subunit alpha | 5 | 2 |
| Isocitrate dehydrogenase [NADP] | 19 | 19 |
| Dihydrolipoyllysine-residue succinyltransferase component of 2-oxoglutarate dehydrogenase complex | 9 | 4 |
| Fumarate hydratase | 15 | 12 |
| **Mitochondrial and Energy Metabolism** | |  |
| Inorganic pyrophosphatase 2 | 15 | 16 |
| ATP synthase subunit alpha | 23 | 20 |
| ATP synthase subunit beta | 26 | 24 |
| ATP synthase subunit O | 13 | 9 |
| ATP synthase-coupling factor 6 | 5 | 6 |
| ATPase | 15 | 14 |
| NADH dehydrogenase [ubiquinone] flavoprotein 2 | 11 | 14 |
| NADH dehydrogenase [ubiquinone] iron-sulfur protein 2 | 13 | 13 |
| NADH-ubiquinone oxidoreductase 75 kDa subunit | 47 | 37 |
| V-type proton ATPase catalytic subunit A | 28 | 26 |
| Low-density lipoprotein receptor-related protein 2;Megalin;Glycoprotein 330 | 20 | 33 |
| Acetyl-Coenzyme A acyltransferase 2 (Mitochondrial 3-oxoacyl-Coenzyme A thiolase) | 26 | 21 |
| Pyruvate dehydrogenase E1 component subunit alpha | 29 | 21 |
| Pyruvate dehydrogenase E1 component subunit beta | 28 | 18 |
| Pyruvate dehydrogenase protein X component | 11 | 12 |
| Succinate dehydrogenase [ubiquinone] flavoprotein subunit | 22 | 17 |
| Succinate dehydrogenase [ubiquinone] iron-sulfur subunit | 7 | 8 |
| Succinyl-CoA:3-ketoacid-coenzyme A transferase 1 | 13 | 10 |
| Creatine kinase | 4 | 4 |
| Creatine kinase B-type;Creatine kinase B chain;B-CK | 11 | 8 |
| Creatine kinase M-type;Creatine kinase M chain;M-CK | 18 | 17 |
| Malate dehydrogenase | 41 | 28 |
| **Amino Acids** |  |  |
| Aspartate aminotransferase | 30 | 29 |
| Glutamate dehydrogenase 1 | 28 | 30 |
| Glutamine synthetase;Glutamate--ammonia ligase | 15 | 18 |
| Glutamine synthetase;Glutamate--ammonia ligase | 16 | 11 |
| Glutathione peroxidase 1;GSHPx-1;Cellular glutathione peroxidase | 10 | 5 |
| Glutathione S-transferase A4 | 14 | 17 |
| Glutathione S-transferase Mu 1 | 32 | 26 |
| Glutathione S-transferase Mu 2 | 18 | 14 |
| Glutathione S-transferase Mu 5;GST class-mu 5;Fibrous sheath component 2 | 17 | 12 |
| Glutathione S-transferase omega-1;GSTO 1-1;p28;Glutathione S-transferase omega-2 | 12 | 11 |
| Glutathione S-transferase P 1 | 18 | 16 |
| Delta-aminolevulinic acid dehydratase;Porphobilinogen synthase | 7 | 11 |
| Dihydrolipoyllysine-residue acetyltransferase component of pyruvate dehydrogenase complex | 18 | 15 |
| L-asparaginase;L-asparagine amidohydrolase;Asparaginase-like protein 1 | 3 | 6 |
| Ornithine aminotransferase | 5 | 10 |
| **Fatty Acid Oxidation** |  |  |
| Acyl-coenzyme A synthetase ACSM2 | 15 | 15 |
| Trifunctional enzyme subunit alpha | 23 | 25 |
| Trifunctional enzyme subunit beta | 23 | 4 |
| Very long-chain specific acyl-CoA dehydrogenase | 31 | 2 |
| Short-chain specific acyl-CoA dehydrogenase | 10 | 3 |
| Acetyl-CoA acetyltransferase | 6 | 6 |
| Carboxylesterase 3;Triacylglycerol hydrolase;Fatty acid ethyl ester synthase | 13 | 4 |
| Carnitine palmitoyltransferase 2 | 12 | 9 |
| Hydroxyacyl-coenzyme A dehydrogenase | 11 | 1 |
| Long-chain specific acyl-CoA dehydrogenase | 17 | 19 |
| Medium-chain specific acyl-CoA dehydrogenase | 21 | 19 |
| Acyl-CoA dehydrogenase family member 10 | 11 | 12 |
| **Urea Cycle** |  |  |
| Carbamoyl Phosphate Synthase I | 18 | 17 |
| Argininosuccinate lyase;Arginosuccinase | 11 | 10 |
| Argininosuccinate synthase;Citrulline--aspartate ligase | 18 | 3 |
| **Others** |  |  |
| Heat shock cognate 71 kDa protein;Heat shock 70 kDa protein 8 | 23 | 20 |
| Peroxiredoxin-1;Thioredoxin peroxidase 2 | 10 | 8 |
| Peroxiredoxin-2;Thioredoxin peroxidase 1 | 5 | 8 |
| Peroxiredoxin-5 | 11 | 9 |
| Peroxiredoxin-6;Antioxidant protein 2;1-Cys peroxiredoxin | 9 | 7 |
| Serotransferrin;Siderophilin;Beta-1 metal-binding globulin | 26 | 16 |
| 60 kDa heat shock protein | 22 | 11 |
| Catalase | 12 | 13 |
| Propionyl-CoA carboxylase alpha chain | 20 | 20 |
| Sulfite oxidase | 11 | 11 |
| Methylmalonate-semialdehyde dehydrogenase [acylating] | 19 | 18 |
| L-lactate dehydrogenase B chain;LDH heart subunit | 15 | 14 |
| L-lactate dehydrogenase;L-lactate dehydrogenase A chain;LDH muscle subunit;Ldha protein | 14 | 15 |
| Acyl-Coenzyme A oxidase 1 | 5 | 5 |
| Methylcrotonoyl-CoA carboxylase beta chain | 9 | 14 |
| Methylcrotonoyl-CoA carboxylase beta chain | 17 | 17 |
| Methylcrotonoyl-CoA carboxylase subunit alpha | 12 | 12 |
| Homogentisate 1 | 18 | 16 |
| Cubilin (Intrinsic factor-cobalamin receptor);Cubilin;Intrinsic factor-cobalamin receptor | 12 | 9 |
| 3-hydroxyisobutyrate dehydrogenase | 11 | 12 |
| 4-trimethylaminobutyraldehyde dehydrogenase | 10 | 10 |
| Aminoacylase-1;N-acyl-L-amino-acid amidohydrolase;ACY-1 | 12 | 4 |
| 2-oxoglutarate dehydrogenase E1 component | 24 | 24 |
| 2-amino-3-carboxymuconate-6-semialdehyde decarboxylase | 6 | 4 |
| Dihydropyrimidinase-related protein 2;ULIP 2 protein | 13 | 12 |
| Citrate synthase | 15 | 12 |
| Dihydrolipoamide branched chain transacylase E2;Lipoamide acyltransferase component of branched-chain alpha-keto acid dehydrogenase complex | 22 | 20 |
| Glycogen phosphorylase | 15 | 21 |
| Hydroxymethylglutaryl-CoA lyase | 7 | 7 |
| Hydroxymethylglutaryl-CoA synthase | 13 | 9 |
